# Evidence of a range expansion in sunfish from 47 years of coastal sightings

**DOI:** 10.1007/s00227-021-04005-8

**Published:** 2022-01-13

**Authors:** Olga Lyashevska, Deirdre Brophy, Steve Wing, David G. Johns, Damien Haberlin, Thomas K. Doyle

**Affiliations:** 1 Marine and Freshwater Research Centre, Galway Mayo Institute of Technology, Galway, Ireland; 2 Cape Clear Bird Observatory, Birdwatch Ireland, Wicklow, Ireland; 3 The Marine Biological Association of the UK, Plymouth, UK; 4 Science Foundation Ireland Research Centre for Energy, Climate and Marine, Environmental Research Centre, University College Cork, Cork, Ireland; 5 School of Biological, Earth and Environmental Sciences, University College Cork, Cork, Ireland

**Keywords:** Mola mola, Jellyfish, Muggiaea atlantica, Dermochelys coriacea, Citizen science, Hurdle model

## Abstract

Almost nothing is known about the historical abundance of the ocean sunfish. Yet as an ecologically and functionally important taxa, understanding changes in abundance may be a useful indicator of how our seas are responding to anthropogenic changes including overfishing and climate change. Within this context, sightings from a coastal bird observatory (51.26$^\circ$ N, 9.30$^\circ$ W) over a 47 year period (from April to October 1971–2017) provided the first long-term index of sunfish abundance. Using a general linear mixed effect model with a hurdle to deal with imperfect detectability and to model trends, a higher probability of detecting sunfish was found in the 1990s and 2000s. Continuous Plankton Recorder (CPR) phytoplankton color indices and the annual mean position of the 13 $^{\circ }$C sea surface isotherm were significantly correlated with the probability of detecting sunfish. An increase in siphonophore abundance (as measured by the CPR) was also documented. However, this increase occurred 10–15 years after the sunfish increase and was not significantly correlated with sunfish abundance. Our results suggest that the observed increase in sunfish sightings is evidence of a range expansion because it was significantly correlated with the mean position of the 13 $^{\circ }$C isotherm which moved northwards by over 200 km. Furthermore, the observed increase in sunfish occured  10 years before sunfish sightings are documented in Icelandic and Norwegian waters, and was concurrent with well-known range expansions for other fish species during the 1990s. This study demonstrates how sustained citizen science projects can provide unique insights on the historical abundance of this enigmatic species.

**Supplementary Information:**

The online version contains supplementary material available at 10.1007/s00227-021-04005-8.

## Introduction

In the past 200 years, humans have witnessed unprecedented changes to the standing animal biomass of our oceans, such that today the biomass of large marine species is only a fraction of former times (Collette et al. 2011; Ferretti et al. 2008). Over-exploitation has brought many fish species to the brink of extinction (e.g., cod in UK and Canada (Rose 2004); southern bluefin tuna; (Commission for the Conservation of Southern Bluefin Tuna 2019)). Unsurprisingly, larger species have fared worse because they are more easily selected by fishing gears, carry a high market value (Sadovy 2001) or may suffer from indirect capture as bycatch (e.g., many shark species) (Dulvy et al. 2014; Hutchings et al. 2010). Ironically, what we know about these fishes is only possible because we harvest them and therefore have a possibility of recording catch statistics (Clarke et al. 2006). For example, the annual demersal fish landings from bottom trawl catches in England and Wales date back to 1888 and represent one of the longest continuous national scale fisheries statistics (Thurstan et al. 2010). However, more typically, up to 70 years of catch statistics and stock assessment data are available for many large species. Indeed, a recent analysis (Hilborn et al. 2020) highlighted the importance of robust abundance data and scientific stock assessments for supporting the recovery of global fish stocks from the historic lows reached in the mid 1990s and maintaining current biomass at or above targeted maximum sustainable yield (MSY) levels. Unfortunately, for some large bodied species, limited or no long-term data exist because they are not directly targeted by fisheries and/or are of little conservation concern, and, therefore, are not assessed. Without such datasets, it is difficult to determine whether they are increasing or decreasing and, therefore, make reasonable conservation assessments and management decisions.

One fish species that has escaped monitoring until very recently is the ocean sunfish, *Mola mola* L. (Nyegaard et al. 2018; Grémillet et al. 2017). This is the largest bony fish in the world (maximum size recorded 3.2 m, Pope et al. 2010) and they are rarely targeted due to their low commercial value with only two known targeted fisheries in Taiwan and Japan (Pope et al. 2010). Furthermore, sunfish are listed as vulnerable by IUCN (Liu et al. 2015), but are not subject to any specific conservation measures with no requirements for their direct monitoring. Yet, very high abundances were documented by recent sunfish aerial surveys in the Atlantic and Mediterranean (Breen et al. 2017; Grémillet et al. 2017). These estimates have led one study (Grémillet et al. 2017) to propose that the abundance of sunfish may be a direct response to an increase in jellyfish. The authors hypothesised that jellyfish have increased due to the combined effects of overfishing and ocean warming (‘rise of slime’ hypothesis, Grémillet et al. 2017). However, without any historical data on jellyfish abundance for these areas it is difficult to draw any conclusions regarding such seemingly high abundances of sunfish. Yet understanding changes in the abundance of sunfish is important because they are ecologically and functionally very different from most fish in the sea. Their size and abundance alone makes them ecologically important but when combined with their unusual diet and high parasite load (up 54 species, Pope et al. 2010), sunfish must also contribute significantly to the functional diversity of our seas.

Here, we describe a unique 47 year database of land-based observations of sunfish that have been recorded during timed ‘sea watches’ by experienced volunteers (birdwatchers) at the Cape Clear Bird Observatory on the southwest coast of Ireland as part of a bird migration monitoring program. Along with sunfish sightings, observers have recorded information of conditions that may affect detectability (time of year, sea state, number of observers), potentially providing a long-term index of the abundance of sunfish in this location in the Celtic Sea. Therefore, the aim of this study is to use this unique data resource to produce a standardised index of the occurrence and relative abundance of sunfish, to examine its temporal variability and to establish relationships with environmental variables. The hypotheses tested were that (1) the number of sunfish sightings increased over time and (2) the observed change in sunfish sightings can be related to changes in food availability (jellyfish abundance) and sea surface temperature.

## Materials and methods

All data preprocessing was done in Python (version 3.7.6, van Rossum 1995) and statistical modeling was done in R (version 4.0.4, R Core Team 2020) using package *lme4* (Bates et al. 2015).

### Sunfish and associated biological and environmental data

Data were collected at the Cape Clear Bird Observatory (51.26$^\circ$ N, 9.30$^\circ$ W, Ireland) as a part of a seabird migration monitoring programme, today co-ordinated by Birdwatch Ireland. Data includes 47 years of observation windows (‘sea watches’) carried out from April through to October between 1971 and 2017 (observations are made throughout the year but sunfish are only sighted during April-October). The monitoring programme was temporarily suspended in 2013–2015. Sea watches were carried out from elevated positions (5–35 m above sea level) on the southern point on the island of Cape Clear. Each observation window consists of a minimum of five minutes of sea watching, where observers scan the sea surface for seabirds. In addition, the observers also record marine megafauna including cetaceans, basking sharks (*Cetorhinus maximus*), leatherback sea turtles (*Dermochelys coriacea*) and ocean sunfish (*Mola spp.*). During sea watches, the following data were documented: effort (duration of sea watch in minutes), number of observers taking part in the sea watch, wind direction (degrees) and sea state (Beaufort scale). In cases when the number of observers was missing but sightings were positive, the number of observers was set to the average for the time series. Records with missing wind direction and sea state were filled with a historical data obtained from the nearby wind stations.

Temperature conditions were described using the annual mean latitude of the 13 $^{\circ }$C isotherm calculated from monthly sea surface temperature (SST) between 48.5$^{\circ }$ N to 52.5$^{\circ }$ N and 12.5$^{\circ }$ W to 4.5$^{\circ }$ W. The 13 $^{\circ }$C isotherm was chosen as it is a good indication of the overall environment experienced by the sunfish in the Northeast Atlantic. Previous studies have used the annual mean SST to derive specific isotherms to examine how they reflect the upper limits of species’ distributions (McMahon and Hays 2006). Location of isotherms was estimated for each longitude/latitude of a 2$^{\circ }$ grid using NOAA Extended Reconstructed SST (Huang et al. 2017).

Indices of sunfish food availability were obtained from the Continuous Plankton Recorder (CPR, area D4) (DOI 10.17031/1695), the most extensive plankton survey in the world (Johns 2021; Richardson et al. 2006). The plankton groups were siphonophores (represented by calycophoran siphonophores) and cnidarians as both are considered important prey items for sunfish (Pope et al. 2010; Nakamura et al. 2015), although prey items such as crustaceans and teleosts appear to be more important for juveniles (Sousa et al. 2016; Phillips et al. 2020). Calycophoran siphonophores were identified by the presence of nectophores. Cnidarians, other than calycophoran siphonophores which are counted separately, were identified by the presence of nematocysts and/or the presence of acellular tissue strewn across the silk (Richardson et al. 2006). The percentage of CPR samples (10 nautical miles or 3 m$^{3}$ of seawater) in which each taxa were recorded was estimated for each year of the time series averaged across 12 months of the year. The two indices of food availability were highly correlated ($\rho$ = 0.84), so only the siphonophore index was used in the subsequent analysis.

The CPR phytoplankton color index (PCI) provided a relative index of phytoplankton abundance (Richardson et al. 2006). The PCI was recorded as 0, 1, 2 or 6.5, representing no green (0) to very dark green (6.5), based on pantone color charts. The PCI was expressed as an annual median. The number of basking sharks and leatherback turtles recorded during each watch were also included in the analysis because both of these species are migratory and feed at a similar trophic level to sunfish (Doyle et al. 2008; Phillips et al. 2020) and, therefore, might represent potential correlates. All continuous variables were expressed as annual means and scaled by subtracting their mean and dividing by standard deviation.

### Modeling trends in sunfish sightings

To evaluate potential relationships between the number of sunfish sighted per minute of effort and explanatory variables, a hurdle model was used (Cragg 1971). This model takes into account the fact that zero sunfish were observed during 86% of sea watches. In this situation, these data are said to be zero-inflated and can be modeled in two steps: the first part of the model includes the zero and non-zero data as a binary response (sunfish presence/absence), the second part models the non-zero data only as a continuous response (the number of sunfish sighted per minute). In step (1), a Bernoulli probability governs the binary outcome of whether a variable has a zero or positive realization. If the realization is positive, the hurdle is crossed. Then, in step (2), the conditional Gamma distribution of the positives is governed by a truncated-at-zero model. Model selection was performed by AIC-based backward variable selection and F-test, using likelihood ratio test to compare resulting models. Data were checked for leverage points, although there were some large values in the dataset, all data fell within the 1.5 interquartile range, and were not considered outliers.

As some of the variables (e.g., duration of the watch, sea state, wind direction) may influence detectability, to isolate their effect, a mixed-effect modeling framework was separately applied to each part of the hurdle model. Two types of explanatory variables were considered: (1) those that are expected to influence detectability and (2) those that might correlate with actual sunfish abundance.

Using this framework, for each part of the hurdle, the factor variable ‘year’ was included as a random effect. Random effects are conditional modes calculated as the difference between the average predicted response for a given set of fixed-effect values (the type (1) variables that may influence detectability) and the response (sunfish presence/absence or abundance) predicted for particular years. These conditional modes were then extracted for each part of the hurdle and were then included as the response variable in a series of general linear models that modeled the effect of the type (2) variables on sunfish occurrence and abundance.

The type (1) variables (Fig. S1) were: the duration of the watch in minutes (watch duration), the number of observers present (observer number), month, sea state and wind direction. Local wind direction was converted from degrees to four cardinal categories: E–NE (0$^{\circ }$–68$^{\circ }$), S–SE (68$^{\circ }$–158$^{\circ }$), W–SW (158$^{\circ }$–248$^{\circ }$) and N–NW (248$^{\circ }$–360$^{\circ }$). The type (2) variables (Fig. S2) described the temperature, feeding conditions for sunfish, phytoplankton color index and the occurrence of other large plankton feeders.

## Results

### Descriptive statistics

#### Sea watch effort

Mean monthly sea watch duration was very consistent over time reaching a maximum of 163 hours per month in the period July-September, and reducing by approximately 50$\%$ in April–June and October. Sea watch effort varied over the years but was above 130 hours for 32 out of the 47 years (68%). The mean number of observers per sea watch was relatively constant throughout the years at 4.6 per year on average (minimum 1.5, maximum 7.4).

#### Conditions during sea watches

The mean sea conditions (as measured by Beaufort wind scale) experienced during sea watches showed no trend over time with most sea watches occurring during sea states of 1–6 (mean 3.9). Similarly, wind direction experienced by the observers during sea watches were consistent over time with a mean wind direction of 210$^\circ$ (Southwest bearing).

#### Mean latitude of 13 $^\circ$C isotherm

The position of the annual mean latitude of the 13 $^\circ$C isotherm varied over time. During the early part of the time series (1970–1980), the mean latitude was 49.3$^\circ$ N; whereas during the latter part of the time series (2000–2010), the mean latitude was 51.5$^\circ$ N which represents a shift of approximately 277 km north (top left, Fig. S2). A notable shift in the position of the mean latitude of the 13 $^\circ$C isotherm occurred during 1990.

#### Indices of food availability

There were very few records of siphonophore nectophores in the early part of the dataset. However, after 2003, there was a sharp rise in siphonophore abundance, with detections every year until the end of the time series and a clear trend of increasing abundance. The maximum abundance of nectophores was recorded in 2003 (0.35 per 3 m$^{3}$ of seawater filtered through the CPR devices). The mean nectophore abundance per decade was 0.002 before 2000, increasing by over 40 times up to 0.09 afterwards.

The PCI expressed as a frequency distribution for each PCI category 0, 1, 2 and 6.5 (bottom middle, Fig. S2) was consistently low with category 0 (no green) being the most frequent in the 1970s. However, there was a gradual increase in greenness with a shift toward 1 in 1985. A high frequency of category 6.5 readings was recorded in 1997, with frequencies in this category returning to a low value in 2007.

#### Basking sharks and leatherbacks

A total of 436 basking sharks (mean of 0.06 per hour of sea watch) and 279 leatherbacks were observed (mean of 0.03 per hour of sea watch) over the entire time series. The highest number of basking sharks was recorded in 1995 (*n* = 74). On average 8.5 basking sharks were sighted per annum. For leatherbacks, most years had very few sightings (mean abundance was 5.47 leatherbacks per annum) but this low level of activity was punctuated with a very sharp increase in leatherbacks sightings in 1989, followed by a peak in sightings in 1993 (*n* = 100) and then a decline from 2000. The highest number of leatherbacks sighted per hour of observation was in August (0.06), and the highest number of sharks was in June (0.18).

#### Sunfish sightings

**Fig. 1:**
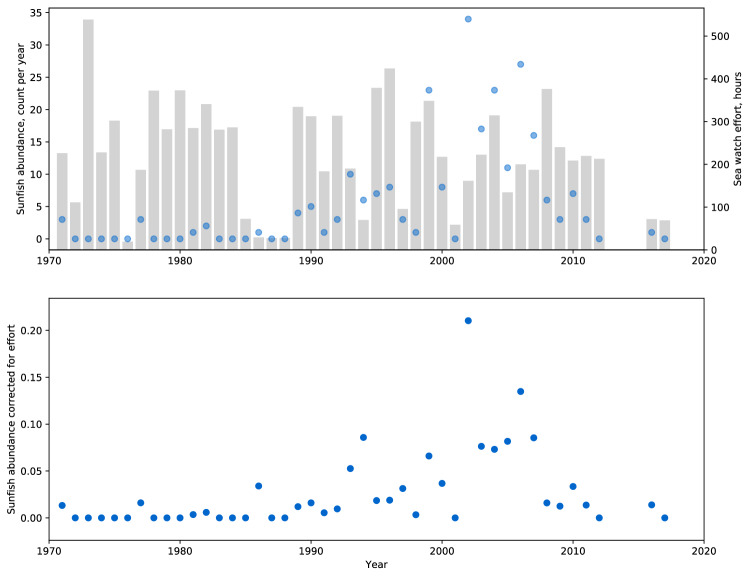
Sunfish abundance per year (blue dots, top panel) superimposed over sea watch effort (gray bars, top panel) and sunfish abundance per hour corrected for effort (bottom panel)

A total of 12% of the observation were collected, while observers were walking or taking a ferry to the island. As these were likely to be recorded only when they were positive, those observations were marked and removed from further analysis. The final dataset contained a total of 204 sunfish sighted over the 47 year time series with a mean of 0.03 (sd 0.24) sunfish per hour of sea watch (Fig. 1).

There was a strong seasonal component to the observations, with most sunfish sighted in the months of August (*n* = 124) and July (*n* = 54), and the least in April and May (*n* = 1). The time series of sunfish sightings, expressed relative to watch duration (sightings per minute), showed that there was a general absence of sightings in the early part of the time series (except for 1971, 1977, 1981 and 1982), but between 1989 and 1998, 3–4 sunfish were sighted per year. From 1999 to 2011 an average of 12–13 sunfish were sighted. The highest count of sunfish in any one year was in 2002 (*n* = 31). There were no sunfish sighted during 2013–2015 which corresponds to when the observatory was closed but there was one in 2016 when it reopened.

### Partitioning out the annual signals

**Fig. 2:**
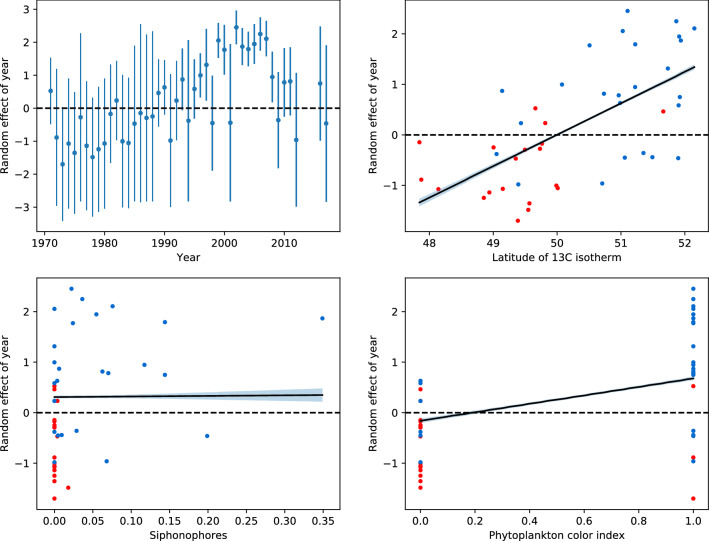
Conditional modes of random effect with an error bar (1.96* standard deviation) around it for each year from the Bernoulli part of the model (top left) and random effect of year against predictors (all others). Red observations are observations before 1990, blue are after 1990. Superimposed lines are predicted partial slopes for a given predictor, when the other predictors were held fixed with a 95% pointwise confidence interval for the fitted values

To partition out the annual signal from the variability in sunfish sightings the type (1) variables were included as fixed effects in a linear mixed effect model. The inclusion of year as a random effect in the Bernoulli part of the hurdle model, which modeled sunfish presence/absence, significantly improved the model’s performance relative to the reduced model showing that there was significant variability between years in the occurrence of sunfish ($\rho < 0.001$). The full model log likelihood was −551.61, the reduced model log likelihood was −617.8. The estimated among-year standard deviation was 1.64. The number of years included in the year random effect was 44, which corresponds to the number of years with complete observations. With the exception of month and wind direction, the fixed effect variables were significant, showing that there was significant intra-annual variability in the occurrence of sunfish (Table S1). The results of the Bernoulli part of the hurdle demonstrated that after accounting for significant effects of observer number, watch duration, sea state, wind direction and month, a considerable increase in probability of sighting a sunfish remains (Fig. 2). The mean annual probability of sighting a sunfish appears to increase up until mid 2000, followed by the decrease in probability towards the end of the time series (2007–2011).

**Fig. 3:**
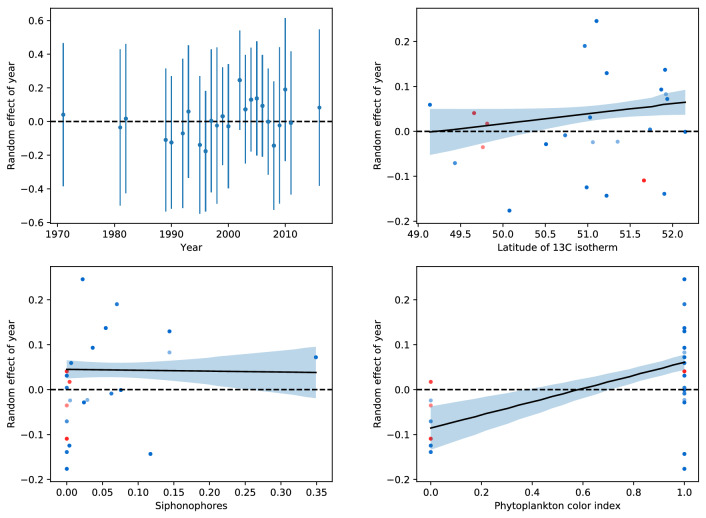
Conditional modes of the random effect with an error bar (1.96* standard deviation) around it for each year from the Gamma part of the model (top left) and random effect of year against predictors (all others). Red observations are observations before 1990, blue are after 1990. Superimposed lines are predicted partial slopes for a given predictor, when the other predictors were held fixed with a 95% pointwise confidence interval for the fitted values

The inclusion of year as a random effect term also improved the fit of the Gamma part of the hurdle model, which modeled the number of sightings per minute for the positive records ($\rho<$0.09). The log likelihood was only marginally different, however. This could be due to the fact that less data were available for the positive sightings. The estimated among-year standard deviation was 0.25. The number of levels in the year random effect was 24, which corresponds to the number of years with positive sightings. Fixed effect variables that were significant were watch duration, month and wind direction W-SW which was the prevalent direction (Table S2). The results of the Gamma part of the hurdle demonstrated that after accounting for significant effects of observer number, watch duration, sea state, wind direction and month, considerable annual variability in abundance remains. Overall, there was no clear trend, but there were higher numbers of positive sightings in the later part of the time series (particularly between 2001 and 2005) (Fig. 3).

Overall, the effects of the type (1) variables on occurrence and abundance had a positive influence (positive slopes of watch duration and observer number in the Bernoulli part) while significant variability on abundance remained. In the Gamma part, the model results indicate that watch duration had a negative influence on the number of sunfish detected and the number of observers had a very small negative effect.

### The effect of covariates

The annual signals in sunfish occurrence and abundance (the conditional modes of the year random effects from each part of the hurdle model) were included as the response variables in a series of general linear models. The aim of the analysis was to determine associations with environmental and biological covariates.

In the Bernoulli part of the hurdle, both PCI and the latitude of the 13 $^\circ$C isotherm were positively and significantly correlated with the probability of sighting a sunfish ($\rho<$0.01). The index of food availability (siphonophores) was also positive, although it did not show any significant correlation with the probability of sighting a sunfish (Table 1).

**Table 1:** GLM summary statistics: Bernoulli model

	Estimate	Std.error	*t* value	$Pr(>|t|)$
(Intercept)	−0.177	0.022	−7.727	1.37e-14 ***
Shark number	0.009	0.013	0.668	0.504
Leatherback number	0.057	0.013	4.149	3.40e-05 ***
PCI	0.926	0.032	28.713	< 2e-16 ***
Siphonophore number	0.009	0.016	0.547	0.585
13 $^\circ$C	0.756	0.016	46.071	< 2e-16 ***

In the Gamma part of the hurdle, PCI, basking sharks and the latitude of the 13 $^\circ$C isotherm were positively correlated with sunfish abundance (see $\rho$-values in Table 2).

**Table 2:** GLM summary statistics: Gamma model

	Estimate	Std.error	*t* value	$Pr(>|t|)$
(Intercept)	−0.096	0.025	−3.855	0.000172 ***
Shark number	−0.007	0.002	−2.911	0.004155 **
Leatherback number	-0.001	0.006	−0.048	0.961722
PCI	0.146	0.026	5.619	9.16e-08 ***
Siphonophore number	−0.001	0.006	−0.203	0.839544
13 $^\circ$C	0.024	0.013	1.831	0.069131

## Discussion and conclusions

What we know about fish abundance mostly comes from fisheries survey data. However, for the largest bony fish in the world, the ocean sunfish (*Mola spp.*), we know almost nothing about its historical abundance as it was rarely targeted. Yet due to its unusual morphology, high fecundity, large size and jellyfish diet (Pope et al. 2010), understanding its past abundance is important as it represents an ecologically and functionally distinct taxon and therefore may be a useful indicator of how our seas are responding to anthropogenic changes including overfishing and climate change (Grémillet et al. 2017).

Within this context, here we provide the first long term index of ocean sunfish abundance derived from observations made from a coastal bird observatory over a 47-year period. The index shows that there was a general absence of sightings before 1989 after which on average 3–4 sunfish were sighted per year (0.031 sunfish per hour) up until 1999. From 2002 to 2007, there was a marked increase in sunfish sightings (21.3 per annum or 0.112 sunfish per hour) where after numbers fluctuated around a lower mean value but with some years of high incidence (e.g., 2010). Using 47 years of presence/absence data, we demonstrated that after accounting for variation in observer number, watch duration, sea state, wind direction and month, there was a higher probability of detecting a sunfish in the 1990s and 2000s than at any other time period at the observatory (top left, Fig. 2). These observations are in agreement with observations from further north in the North East Atlantic (NEA) where increases in sunfish sightings have been documented. For example, in Icelandic waters, Palsson and Astthorsson (2017) recorded an increase in the occurrence of sunfish since the 2000s and Frafjord et al. (2017) reported that annual records of more than five sunfish in Norwegian waters all occurred after 2000. Such observations were reported as evidence of a range expansion for ocean sunfish. Here, we provide much stronger evidence for a range expansion from a coastal observatory where experienced bird watchers recorded an abrupt change in abundance of sunfish during the 1990s followed by a larger increase in the 2000s.

Several lines of evidence suggest that the observed increase in sunfish in the 1990s and 2000s represents a range shift in this species. (1) Sunfish are ectotherms, have a preferred temperature range between 13 and 23 $^\circ$C and are highly migratory species that are known to migrate north during the spring months into temperate waters tracking a strict thermal envelope of sea surface temperatures (Sousa et al. 2016; Sims et al. 2009). As such, the northerly extent of their summer range is likely determined by temperature. (2) The annual mean position of the 13 $^\circ$C isotherm in the 1970s and 1980s was located $\sim$200 km south of Ireland but after 1990 there is a dramatic and rapid northerly shift in its position (Fig. S3). This shift in isotherms would make Irish waters more favourable for sunfish and here we show that the latitude of this isotherm was significantly correlated with the probability of detecting a sunfish (top right, Fig. 2). (3) A well-known climate-induced ecosystem change occurred across the NEA in the 1990s with numerous studies documenting range expansions for fish species (Alheit et al. 2019). For example, red mullet *Mullus surmuletus* and sea bass *Dicentrarchus labrax* increased in the English Channel and spread into central North Sea (Brander et al. 2003) and large increases in anchovies and sardines catches were reported in the North Sea in the 1990s and mid-1990s respectively (Beare et al. 2004; Alheit et al. 2012). Such changes were observed for other taxa too. For example, a rapid northwards expansion of the endangered Balearic shearwater (*Puffinus mauretanicus*) was associated with a 0.6 $^\circ$C sea surface temperature increase in the mid-1990s (Wynn et al. 2007) and with associated changes in plankton and their prey fish species (Luczak et al. 2011). More broadly in the NEA, there has been a poleward movement of warm water copepod species associated with a retraction northwards in a number of subarctic and arctic copepod species in the north (Beaugrand et al. 2002). Many of these biological changes have been associated with a switch from a negative to a positive phase of the Atlantic Multidecadal Oscillation (AMO) in the 1990s (Alheit et al. 2019; Hatun et al. 2009). The AMO influences major currents in the NEA such as the Shelf Edge Current which during the positive phase pushes waters with Lusitanian biomes much further north (Hatun et al. 2009). Taken together, all these lines of evidence suggest that our increased probability of sighting sunfish is more parsimonious with a range expansion of sunfish rather than an increase in numbers.

The broken effort in the last seven years of the observatory makes it difficult to determine if numbers have remained low, decreased or even increased, however other evidence suggests sunfish were very abundant in Irish waters during this period. For example, during 2015–2016, large-scale offshore aerial surveys in Irish waters estimated >12,000 sunfish during the summer months, or a density of 4.3 individuals per 100 km^2^ flown (Breen et al. 2017). Similar surveys were also carried out in the southeast Celtic Sea and the Bay of Biscay in 2011–2012 with higher densities reported (Grémillet et al. 2017). So rather than a decline, it may be that local conditions at the observatory are less favorable for sunfish (e.g., changes in local hydrography) and therefore, fewer sunfish are detected. Local changes may also alter sunfish behavior so that they are less visible at the surface. For example, in warmer waters sunfish may require less time basking at the surface to rewarm (Nakamura et al. 2015). However, comparison of sunfish sightings with leatherback sea turtle sightings at the observatory may offer some additional insight. For example, leatherback numbers at the observatory peaked in the 1990s (Fig. S2) and were lower in the 2000s and 2010s and were signficantly correlated with sunfish numbers (Table 1). This pattern agrees with Botterell et al. (2020) who examined all leatherback sightings and strandings for the UK and Ireland and documented a decline in leatherback numbers during the 2000s and then again from 2010 onwards. This agreement suggests that the lower sunfish sightings during the last seven years at the observatory may be real and not due solely to the broken and/or reduced effort at the observatory. Continued monitoring, ideally across a wider network of coastal observatories is needed address this question. Such coastal observatory data from networks could be combined with regular aerial survey data and telemetry studies to refine our understanding of the process of range expansion in this tractable species (Pecl et al. 2017).

One question we set out to address was whether sunfish have benefited from the ‘rise of slime’, i.e., the hypothesis that jellyfish have increased due to the combined effects of overfishing and ocean warming (Grémillet et al. 2017). A lack of both jellyfish and sunfish historical abundance data has made it almost impossible to say whether the two are linked but using a 47 year time series for both, here we show the effect of siphonophore abundance on sunfish presence/absence to be positive but not significant. Clearly there is a dramatic increase in siphonophores recorded in the 2000s but importantly, this increase occurs 10–15 years after the sunfish increase. This may be because species respond differently to climate change (Pecl et al. 2017) and as a highly migratory species capable of moving 30 km a day (Potter et al. 2011), sunfish can respond more immediately to local changes and can move further north if conditions are favorable. In contrast, siphonophores as plankton, may be stuck within a water mass and will move northwards with the isotherms as their realised niche shifts. However as a recent study has shown, some plankton display niche plasticity and can adapt to local changes of SST and, therefore, move more slowly northwards (Chivers et al. 2017).

The dramatic increase in calycophoran siphonophores is by itself a notable finding as there are limited time series for gelatinous zooplankton available over such long time periods and over such a broad scale. Clearly, there are issues on how well the CPR samples gelatinous zooplankton (Baxter et al. 2010) but the size and distinct morphology of calycophoran siphonophore nectophores makes them a good candidate for detecting trends in gelatinous zooplankton. A recent study in the Celtic Sea has shown that *Muggiaea atlantica* is the most likely calycophoran siphonophore sampled by the CPR but two physoconect siphonophores were much more abundant and may be sampled too (Haberlin et al. 2019). Our findings support previous studies that have documented increases and a range expansion for *M. atlantica* in the Western English Channel and the east coast of Scotland (Blackett et al. 2016). The timing of this increase in Scotland coincides with the dramatic increase documented here in the Celtic Sea. It is possible that these siphonophore increases are related to changes in the AMO which switched from a negative to a positive phase in the mid-1990s. This switch to a positive phase resulted in large scale changes in the strength and direction of several major currents in the NEA, including the Shelf Edge Current which is thought to have strengthened (Hatun et al. 2009; Alheit et al. 2019) and, therefore, may be playing an important role in driving the observed increased in siphonophores.

In the absence of any long-term fisheries capture (or bycatch data) in the NEA for ocean sunfish, reconstruction of their past trends in abundance must rely on sightings records, which are collected opportunistically (Palsson and Astthorsson 2017; Frafjord et al. 2017) or as part of monitoring surveys for other species and through citizen science initiatives. However, such data are more prone to sources of imprecision and bias than data collected through rigorous scientific surveys (Bird et al. 2014). Here, we present a framework for the appropriate statistical treatment of these data to account for inherent bias and to effectively detect the underlying long-term trends in the time series.

## Supplementary Information

Below is the link to the electronic supplementary material.

Supplementary file1 (PDF 1,5439 KB)

Supplementary file2 (JPG 2,459 KB)

## Data Availability

The datasets are available from the corresponding author on reasonable request.

## Declarations

### Code availability

The code used in the data processing and analysis is available in the github repository https://github.com/lyashevska.
