# Supplementary material for: Evidence of a range expansion in sunfish from 47 years of coastal sightings
Source: Mar Biol. 2022 Jan 13;169(2):20. doi: 10.1007/s00227-021-04005-8 (PMC8827117; doi:10.1007/s00227-021-04005-8)
Supplement: Supplementary file 1 — Supplementary file1 (PDF 1,5439 KB) [file 227_2021_4005_MOESM1_ESM.pdf]

Evidence of a range expansion in sunfish from 47 years of coastal sightings

Olga Lyashevska[a], Deirdre Brophy[a], Steve Wing[b], David G. Johns[c], Damien Haberlin[d], Thomas K. Doyle[d,e,*]

[a] *GMIT, Marine and Freshwater Research Centre, Ireland*
[b] *Birdwatch Ireland*
[c] *The Marine Biological Association of the UK*
[d] *Science Foundation Ireland Research Centre for Energy, Climate and Marine, Environmental Research Centre, University College Cork, Ireland*
[e] *School of Biological, Earth and Environmental Sciences, University College Cork, Ireland*

1. Supplementary Material

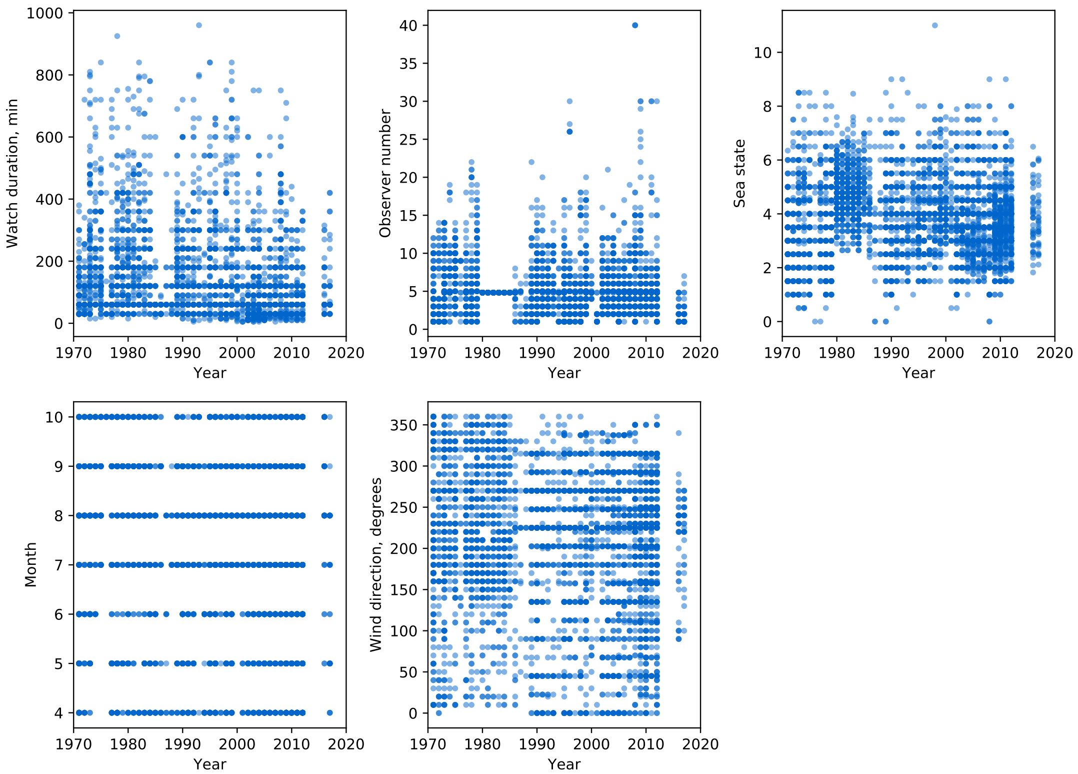

Fig. S1 Time series of type (1) variables that are expected to influence detectability. Each dot represents an individual observation, due to the large number of points some points overplot, darker blue indicates the intensity of overplotting.

*Corresponding author
Email addresses: `Olga.Lyashevska@gmit.ie` (Olga Lyashevska), `Deirdre.Brophy@gmit.ie` (Deirdre Brophy), `steve.ccbo@gmail.com` (Steve Wing), `djoh@mba.ac.uk` (David G. Johns), `D.Haberlin@ucc.ie` (Damien Haberlin), `T.Doyle@ucc.ie` (Thomas K. Doyle)

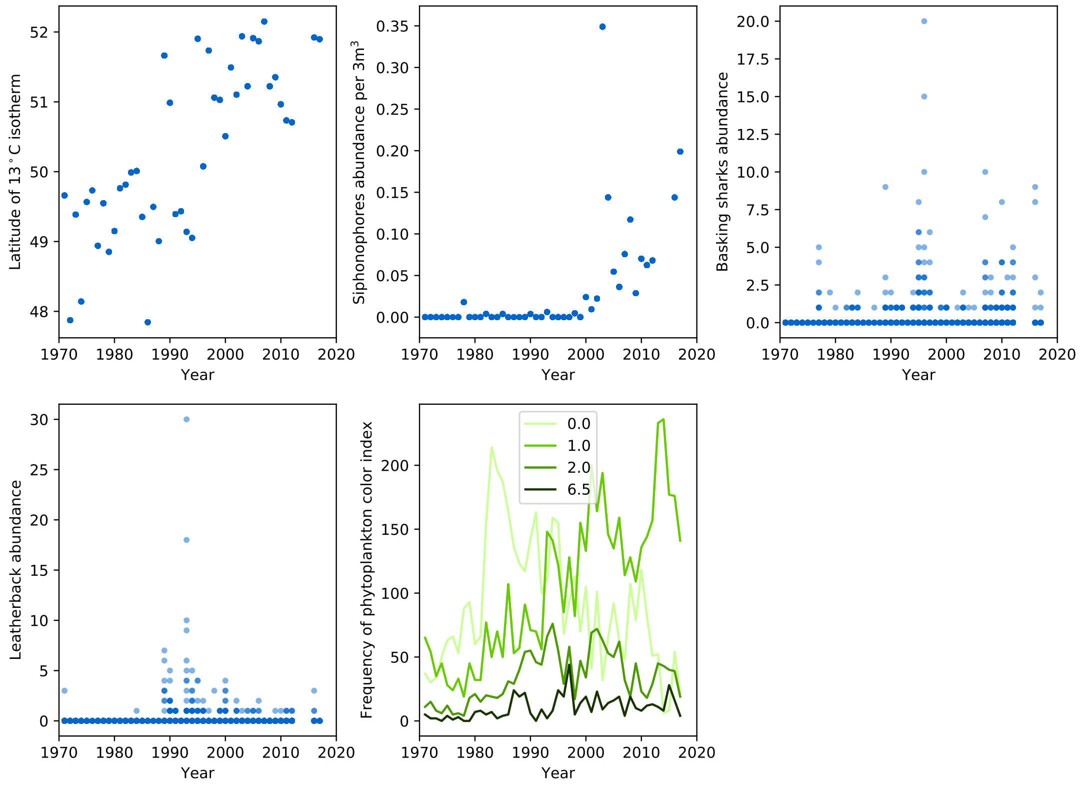

Fig. S2 Time series of type (2) variables that were identified as possible correlates with actual sunfish abundance. Each dot in abundance plots represents an individual observation per day, in all other plots it is an annual value. Due to the large number of points in abundance plots some points overplot, darker blue indicates the intensity of overplotting.

Table S1 Fixed effects summary statistics: Bernoulli model

	Estimate	Std.Error	z value	$Pr(>\|z\|)$
(Intercept)	-4.8464	0.6604	-5.824	5.76e-09 ***
watch duration	-1.5852	0.0750	7.801	6.13e-15 ***
month	-1.0866	0.0620	1.397	0.162562
observer number	-1.2009	0.0782	2.569	0.010194 *
wind direction (N-NW)	-1.3691	0.2769	-1.333	0.182577
wind direction (S-SE)	-1.2405	0.3174	-0.758	0.448657
wind direction (W-SW)	-1.4197	0.2761	-1.520	0.128482
sea state	-1.2645	0.0733	-3.609	0.000307 ***

Table S2 Fixed effects summary statistics: Gamma model

	Estimate	Std.Error	z value	$Pr(>\|z\|)$
(Intercept)	-2.876	0.761	-4.993	5.94e-07 ***
watch duration	-0.539	0.435	-12.371	$<$ 2e-16 ***
month	-0.183	0.701	-2.615	0.00892 **
observer number	-0.029	0.559	-0.533	0.59388
wind direction (N-NW)	-0.156	0.475	-1.061	0.28848
wind direction (S-SE)	-0.169	0.740	-0.974	0.33030
wind direction (W-SW)	-0.317	0.478	-2.149	0.03161 *
sea state	0.004	0.467	0.093	0.92593

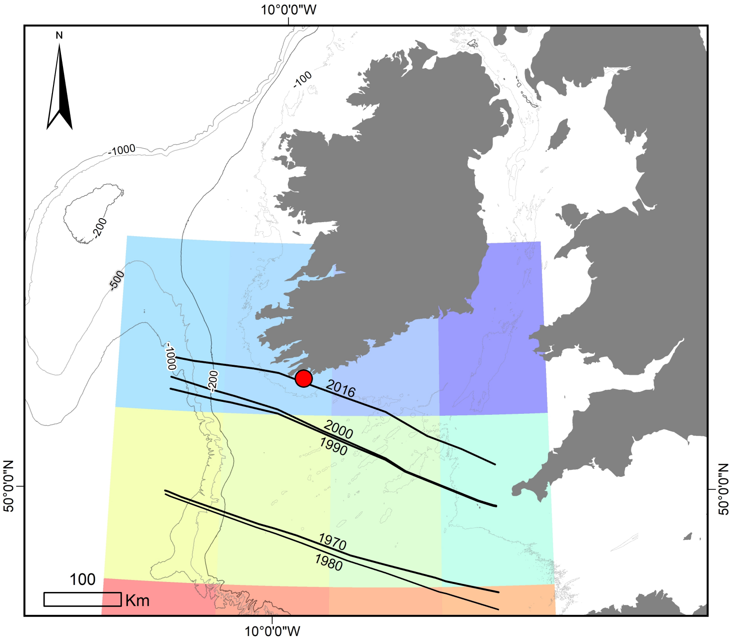

Fig. S3 The annual mean position of the 13°C isotherm. Colours represent the mean SST temperature raster layer on a 2x2 degree grid for 2016. Red marker is a position of the Cape Clear Bird Observatory.
