# Supplementary figures and images for: Evidence of a range expansion in sunfish from 47 years of coastal sightings
Source: Mar Biol. 2022 Jan 13;169(2):20. doi: 10.1007/s00227-021-04005-8 (PMC8827117; doi:10.1007/s00227-021-04005-8)

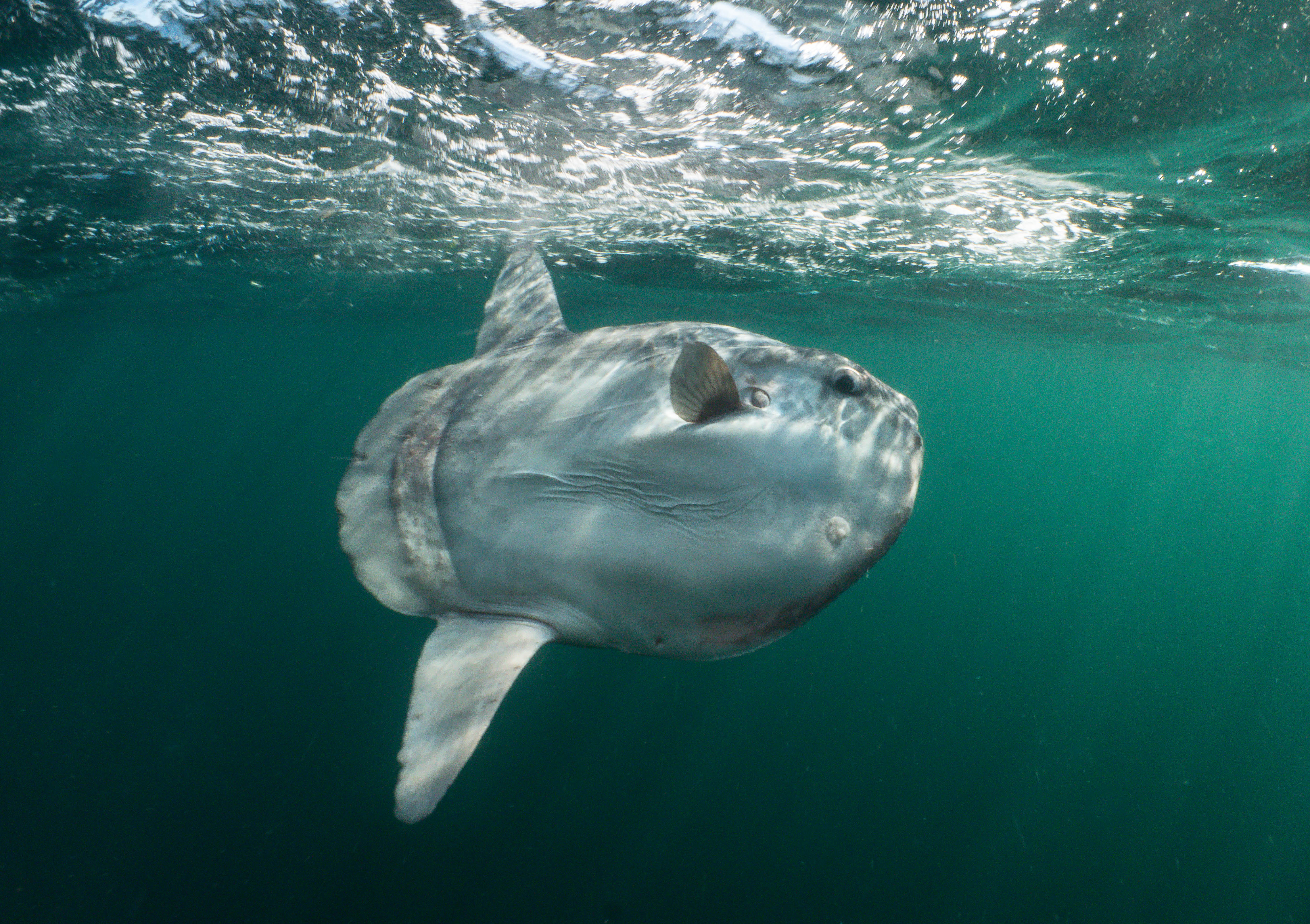

Supplement: Supplementary file 2 — Supplementary file2 (JPG 2,459 KB) [file 227_2021_4005_MOESM2_ESM.jpg]
